# Peer Review of “Health Care System Overstretch and In-Hospital Mortality of Intubated Patients With COVID-19 in Greece From September 2020 to April 2022: Updated Retrospective Cohort Study”

**DOI:** 10.2196/59639

**Published:** 2024-06-10

**Authors:** Anonymous

**Keywords:** COVID-19, pandemic, health care disparities, intensive care unit, ICU, right to health, quality of care, intubation, mortality, health disparity, health inequality, surveillance data, in-patient, mortality, COVID-19 patient, hospitalization, disparity, inequality, surveillance, health care system, Greece, region, Delta, Omicron, vaccination, vaccine, public health, patient load, deterioration, time

*This is the peer review report for “Health Care System Overstretch and In-Hospital Mortality of Intubated Patients With COVID-19 in Greece From September 2020 to April 2022: Updated Retrospective Cohort Study.”*

## Round 1 Review

### General Comments

This paper [1] is interesting but needs improvement.

### Specific Comments

#### Major Comments

*We aimed to update this analysis to include the large “Delta” and “Omicron” waves that affected Greece during 2021‐2022*

So why did you analyze data also from 2020?

*Vaccination did not affect the mortality of these already severely ill patients.*

Did you check how many months before they had received the vaccine? How many of the participants were not vaccinated?

*Vaccination did not show a statistically significant association with mortality, regardless of the number of doses received (Figure 2).*

This needs rephrasing if all participants were vaccinated at least once.

*Between 1 September 2020 and 3 April 2022...*

I see that your previous study included data up to May 2021. Thus, there is a lot of overlap in the data of these two studies.

There are many errors in the use of English and typos which makes it difficult to understand. See examples below:

*Mortality was significantly higher above 400 patients*

This is not very clear (please rephrase).

*...with an adjusted Hazard Ratio of 1.22, 95% CI: 1.09‐1.38)*

Where does the parenthesis open?

*...rising progressively up to 1.48 (95% CI: 1.31‐1.69) for 800+ patients.*

This is not clear.

*...we found no statistically association between mortality and...*

Please correct typo.

#### Minor Comments

It would be good to include DOIs to all references.
